# Variations of parathyroid hormone and bone biomarkers are concordant only after a long term follow-up in hemodialyzed patients

**DOI:** 10.1038/s41598-017-12808-3

**Published:** 2017-10-03

**Authors:** Pierre Delanaye, Xavier Warling, Martial Moonen, Nicole Smelten, François Jouret, Jean-Marie Krzesinski, Nicolas Maillard, Hans Pottel, Etienne Cavalier

**Affiliations:** 1 Division of Nephrology-Dialysis-Transplantation, University of Liège, CHU Sart Tilman (ULg CHU), Liège, Belgium; 2 Division of Nephrology-Dialysis, Centre Hospitalier Régionale (CHR) « La Citadelle », Liège, Belgium; 3 Division of Nephrology-Dialysis, Centre Hospitalier Bois de l’Abbaye et de Hesbaye (CHBAH), Seraing, Belgium; 4 Division of Nephrology-Dialysis-Transplantation, Hôpital Nord, University Jean Monnet, Saint Etienne, France; 5 Division of Public Health and Primary Care, KU Leuven Campus Kulak Kortrijk, Kortrijk, Belgium; 6 Division of Clinical Chemistry, University of Liège (ULg CHU), Liège, Belgium

## Abstract

End-stage renal disease is associated with mineral and bone disorders. Guidelines recommending therapies should be based on serial assessments of biomarkers, and thus on variations (Δ), rather than scattered values. We analyzed the correlations between ΔPTH and Δbone biomarkers such as bone-specific alkaline phosphatase (b-ALP), Beta-CrossLaps (CTX), osteocalcin, intact serum procollagen type-1 N-propeptide (P1NP), and tartrate-resistant acid phosphatase 5B (TRAP-5B) at different time-points. In this prospective observational analysis, variations of biomarkers were followed after 6-week (n = 129), 6-month (n = 108) and one-year (n = 93) period. Associations between variations were studied by univariate linear regression. Patients followed for one-year period were classified (increaser or decliner) according to variations reaching the critical difference. Over the 6-week period, only ΔCTX was correlated with ΔPTH (r = 0.38, p < 0.0001). Over the one-year period, correlations between ΔPTH and Δbone biomarkers became significant (r from 0.23 to 0.47, p < 0.01), except with ΔTRAP-5b. Correlations between Δbone biomarkers were all significant after one-year period (r from 0.31 to 0.68, p < 0.01), except between Δb-ALP and ΔTRAP-5b. In the head-to-head classifications (decliners/increasers), the percentage of concordant patients was significantly higher over the one-year than the 6-week period. A concordance between ΔPTH and Δbone biomarkers is observed in dialysis patients, but only after a long follow-up.

## Introduction

Chronic Kidney Disease (CKD) is associated with mineral and bone disorders (MBD). If bone health includes important concepts such as bone mineralization and bone volume, abnormalities in bone turnover remain key and specific to the dialysis population^1^. The gold standard for bone turnover diagnosis is bone biopsy^2^. However, such an invasive technique is difficult to apply in daily practice in every patient. Also, it is cumbersome to repeat such biopsies for bone turnover monitoring^3^. Therefore, nephrologists rely on bone biomarkers to monitor CKD-MBD^1^. The recent 2017 Kidney Disease: Improving Global Outcomes (KDIGO) clinical practice guidelines recommend to measure serum parathyroid hormone (PTH) and bone-specific alkaline phosphatase (b-ALP)^4,5^. However, the interpretation of these two parameters is not easy, notably because of lack of standardization in the dosages^6–8^. In cross-sectional studies, a good correlation is found between the two biomarkers^3,9–11^. However, their performance to predict bone turnover assessed by bone biopsies in transversal studies remains actually disappointing^3,12–14^. For this reason, the revised 2017 KDIGO guidelines suggest that potential CKD-MBD therapies should be based on serial assessments of biomarkers, and thus on trends or variations (Δ), more than on one-single transversal result. In a previous work, we suggested that the correlations between variations of PTH (ΔPTH) and b-ALP (Δb-ALP) were poor^11^. This study was however retrospective and sample size was limited. In the current work, we prospectively followed variations of PTH and other bone biomarkers on a 6-week, 6-month and one-year period.

## Materials and Methods

Serum PTH and bone biomarker concentrations were measured in hemodialysis patients from three independent hospitals in Liège (Belgium) and surrounding areas (Centre Hospitalier Universitaire du Sart Tilman, Centre Hospitalier Regional de La Citadelle, Centre Hospitalier du Bois de l’Abbaye de Seraing) in 2013. All procedures performed in the current study were in accordance with the ethical standards of the institutional research committee and with the 1964 Helsinki declaration and its later amendments or comparable ethical standards. The protocol was approved by the Ethics Committee of our institution “Comité d’éthique hospitalo-facultaire universitaire de Liège” (Belgian number study: B707201215885). Informed signed consent was obtained from all participants.

Samples were drawn in participating patients at baseline, then after 6 weeks, 6 months, and one year. Blood samples were collected before the first dialysis session of the week. Samples were immediately centrifuged and kept frozen at −80 °C until determination. Third-generation PTH (DiaSorin-Liaison, Stillwater, MN), intact serum procollagen type 1 N propeptide (P1NP), tartrate-resistant acid phosphatase 5B (TRAP-5B), osteocalcin, and Beta-CrossLaps (CTX)(IDS-iSYS, Boldon, UK) were measured in the same batch and in the same laboratory (department of Clinical Chemistry, University of Liège) accredited for the ISO 15189 Guideline. Inter-assay coefficients of variation were below 10% for all measurements^15^. Serum calcium, phosphorus, C-reactive protein and albumin were also measured at baseline and after one-year period (Roche Cobas, Mannheim, Germany).

Data are expressed as mean ± standard deviation (SD) when distribution was normal and as median with interquartile range [IQR] when not. Normality was assessed by the Shapiro-Wilk test. In the transversal analysis, Pearson’s coefficients of correlation were calculated between PTH and bone biomarkers at baseline and after one-year. Then, three different analyses were done to study the variations of PTH and bone biomarkers over time. First, coefficients of correlation were calculated focusing on the variations of PTH (ΔPTH, in %) and bone biomarkers (Δbone biomarkers, in %). Correlations between ΔPTH and Δbone biomarkers over a 6-week, 6-month and one-year period were thus calculated. Second, the concept of critical difference (CD) or least significant change was introduced in the analysis. In the field of Clinical Chemistry, CD represents the threshold from which a relative change between two consecutive measurements in the same patient is considered as significant. CD was previously determined for PTH, b-ALP, P1NP and TRAP-5b in the dialysis context^15^: 43%, 23%, 32% and 24%, respectively. As an example, a relative difference between two PTH consecutive results in the same patient can be considered as clinically relevant if it reached 43%. Patients were then classified according to these significant variations (stable, increaser or decliner). Classifications were compared by exact χ². Third, the slopes of different biomarkers were compared over one-year period (using the 4 available concentrations). Slopes were built and compared by simple linear regression and Pearson correlation. A p value of 0.008 was considered as significant after Bonferroni correction.

## Results

### Characteristics of the population

One hundred thirty-one patients signed the informed consent. Two patients were excluded from the analysis because of extreme PTH variations (from 36 to 1140 and from 1020 to 67 pg/mL). Median age was 73.3 [18.1] year-old and 55.8% were men. During the follow-up, 36 patients dropped-out the analysis because of death (n = 18), loss of follow-up (n = 8), renal transplantation (n = 8) or hospitalization (n = 2). The total sample at baseline, after 6-week, 6-month and one-year was 129, 123, 108 and 93, respectively. Clinical and biological characteristics at baseline and over one-year period are summarized in Table 1.

**Table 1:** Clinical and biological characteristics of the patients at baseline and after one-year follow-up.

	Baseline	1-year
Sample	129	93
Age	73.3 [18.1]	74.1 [16.5]
Gender (% men)	55.8%	53.8%
Dry weight (kg)	71 [25]	71 [22]
Height (m) (n = 116)	1.63 ± 0.10	1.62 ± 0.10
BMI (kg/m²) (n = 116)	25.6 [9.9]	25.5 [9.9]
Diabetes (%)	42.2%	41.9%
Hypertension (%)	88.3%	85.0%
Dialysis vintage (month)	25.0 [39.0]	25.0 [39.0]
Therapy (%)		
Calcium-based chelator	55.8%	50.0%
Non-Calcium based chelator	47.2%	43.6%
Native vitamin D	85.3%	80.4%
Active vitamin D	20.2%	29.8%
Cinacalcet	15.5%	23.4%
Serum calcium (mmol/L)	2.21 ± 0.17	2.20 ± 0.18
Serum phosphate (mmol/L)	1.55 [0.68]	1.49 [0.64]
Albumin (g/L)	39 ± 5	39 ± 5
C-reactive protein (mg/L)	4.8 [8.6]	4.2 [6.0]
25-OH vitamin D (ng/mL)	27.9 [24.6]	26.5 [23.3]
1,25 vitamin D (pg/mL)	18.4 [12.0]	14.7 [12.1]
PTH (pg/mL)	166 [175]	179 [216]
b-ALP (µg/L)	23.4 [17.9]	27.4 [31.3]*
CTX (ng/L)	2211 [2605]	1870 [2525]
Osteocalcin (ng/mL)	155 [194]	200 [270]
P1NP (ng/mL)	71 [62]	87 [106]*
TRAP-5b (U/L)	4.4 [2.9]	4.0 [3.5]

BMI: body mass index, *p < 0.05 between baseline and one-year result.

### Pearson correlations

In the transversal analysis, a significant positive correlation was found between PTH and different bone biomarkers (except no correlation with TRAP-5B), both at baseline (r ranging from 0.42 to 0.72, p < 0.0001) and after one-year follow-up (r ranging from 0.51 to 0.72, p < 0.0001) (Table 2). Significant positive correlations were also found between the different bone biomarkers at baseline (r ranging from 0.22 to 0.81, p < 0.01) and after one-year follow-up (r ranging from 0.22 to 0.82, p < 0.01).

**Table 2:** Coefficient of correlation between biomarkers at baseline (Italic) and after one-year follow-up (bold).

	PTH	b-ALP	CTX	osteocalcin	P1NP	TRAP-5b
PTH	XXXXX	**0.62** ***P*** < ***0.0001***	**0.72** ***P*** < ***0.0001***	**0.71** ***P*** < ***0.0001***	**0.51** ***P*** < ***0.0001***	**0.10 NS**
b-ALP	*0.57* *P* < *0.0001*	XXXXX	**0.75** ***P*** < ***0.0001***	**0.61** ***P*** < ***0.0001***	**0.78** ***P*** < ***0.0001***	**0.22** **P = 0.0332**
CTX	*0.72* *P* < *0.0001*	*0.72* *P* < *0.0001*	XXXXX	**0.82** ***P*** < ***0.0001***	**0.79** ***P*** < ***0.0001***	**0.44** ***P*** < ***0.0001***
Osteocalcin	*0.72* *P* < *0.0001*	*0.70* *P* < *0.0001*	*0.81* *P* < *0.0001*	XXXXX	**0.53** ***P*** < ***0.0001***	**0.25** **P = 0.0157**
P1NP	*0.42* *P* < *0.0001*	*0.73* *P* < *0.0001*	*0.75* *P* < *0.0001*	*0.61* *P* < *0.0001*	XXXX	**0.52** ***P*** < ***0.0001***
TRAP-5b	*0.06 NS*	*0.32* *P* = *0.0002*	*0.30* *P* = *0.0005*	*0.22* *P* = *0.0105*	*0.65* *P* < *0.0001*	XXXXX

NS: not significant. Significant results in *italic*.

By contrast, when the variations of concentrations are considered (ΔPTH and Δbone biomarkers), associations were much less solid than in the transversal analyses. Over the 6-week period (Table 3), only CTX was correlated with ΔPTH (r = 0.38, p < 0.0001). Also, several correlations between Δbone biomarkers were not significant. However, over the 6-month period (Table 3) and still more for the one-year period (Table 3), correlations between ΔPTH and Δbone biomarkers became significant (r ranging from 0.23 to 0.47, p < 0.01 after one year), except for the absence of correlation between ΔPTH and ΔTRAP-5b. Also, correlations between Δbone biomarkers were all significant (and stronger than between ΔPTH and Δbone biomarkers) after one-year period (r ranging from 0.31 to 0.68, p < 0.01 after one year), except that no correlation existed between Δb-ALP and ΔTRAP-5b.

**Table 3:** Coefficient of correlation between variations (Δ) of biomarkers between baseline and 6-week (n = 123), 6-month (n = 108) and one-year (n = 93).

6-week n = 123	ΔPTH	Δb-ALP	ΔCTX	Δosteocalcin	ΔP1NP	ΔTRAP-5b
ΔPTH	XXXXX					
Δb-ALP	−0.08 NS	XXXXX				
ΔCTX	*0.38* *P* < *0.0001*	0.07 NS	XXXX			
ΔOsteocalcin	−0.03 NS	*0.31* *P* = *0.0004*	0.15 NS	XXXXX		
ΔP1NP	−0.14 NS	*0.43* *P* < *0.0001*	0.06 NS	*0.52* *P* < *0.0001*	XXXX	
ΔTRAP-5b	0.04 NS	*−0.26* *P* = *0.0036*	*0.20* *P* = *0.0247*	−0.07 NS	−0.06 NS	XXXXX
6-month n = 108	**ΔPTH**	**Δb-ALP**	**ΔCTX**	**Δosteocalcin**	**ΔP1NP**	**ΔTRAP-5b**
ΔPTH	XXXXX					
Δb-ALP	0.11 NS	XXXXX				
ΔCTX	*0.39* *P* < *0.0001*	*0.38* *P* < *0.0001*	XXXX			
ΔOsteocalcin	*0.27* *P* = *0.0049*	*0.26* *P* = *0.007*	*0.32* *P* = *0.0008*	XXXXX		
ΔP1NP	0.07 NS	*0.44* *P* < *0.0001*	*0.48* *P* < *0.0001*	*0.54* *P* < *0.0001*	XXXX	
ΔTRAP-5b	0.04 NS	0.03 NS	*0.27* *P* = *0.0045*	0.07 NS	*0.24* *P* = *0.013*	XXXXX
One-year n = 93	**ΔPTH**	**Δb-ALP**	**ΔCTX**	**Δosteocalcin**	**ΔP1NP**	**ΔTRAP-5b**
ΔPTH	XXXXX					
Δb-ALP	*0.29* *P* = *0.0054*	XXXXX				
ΔCTX	*0.47* *P* < *0.0001*	*0.44* *P* < *0.0001*	XXXX			
ΔOsteocalcin	*0.36* *P* = *0.0004*	*0.41* *P* < *0.0001*	*0.43* *P* < *0.0001*	XXXXX		
ΔP1NP	*0.40* *P* = *0.0001*	*0.68* *P* < *0.0001*	*0.59* *P* < *0.0001*	*0.65* *P* < *0.0001*	XXXX	
ΔTRAP-5b	0.08 NS	0.14 NS	*0.36* *P* = *0.0004*	*0.31* *P* = *0.0021*	*0.37* *P* = *0.0003*	XXXXX

NS: not significant. Significant results in *italic*.

### Critical difference

In this analysis, only the patients who fulfilled the entire one-year period (n = 93) were included. According to the respective CD of markers, the patients were classified as “stable”, “increaser” or “decliner”, at 6-week and one-year. As expected, the percentage of stable patients was higher after 6-week than one-year for all biomarkers (exact χ², p < 0.05), except for TRAP-5b. Head-to-head classifications (stable/decliners/increasers) of different biomarkers are given over the 6-week and one-year period in Table 4. Globally, biomarkers similarly classified the patients in 38 to 72% of patients. Severe discordances (meaning that one biomarker significantly increased whereas the other actually significantly decreased) were found in 1 to 13% of patients. Results of head-to-head comparisons including TRAP-5b were systematically less good than with other bone biomarkers. Concordances in classifications were slightly better between b-ALP and CTX than between PTH and b-ALP or PTH and intact P1NP. Concordances over a six-week period are better than over one-year period, but this analysis must be adjusted by the much higher percentage of stable patients in the 6-week period. Excluding stable patients and focusing on increasers and decliners, the percentage of concordant patients (i.e. the percentage of patients classified as decliners or increasers) was significantly higher over the one-year period in the head-to-head comparisons of biomarkers.

**Table 4:** Head-to-head comparison of biomarkers variations (increaser/stable/decliner) according to their respective critical difference.

6-week (n = 93)	Increased b-ALP (Δb-ALP > + 23%)	b-ALP stable (Δb-ALP within 23%)	Decreased b-ALP (Δb-ALP > −23%)
Increased PTH (ΔPTH > + 43%)	**1 (1%)**	13 (14%)	*1 (1%)*
PTH stable (ΔPTH within 43%)	14 (15%)	**53 (56%)**	*1 (1%)*
Decreased PTH (ΔPTH > −43%)	*4 (4%)*	4 (4%)	**2 (2%)**
One-year (n = 93)	Increased b-ALP (Δb-ALP > + 23%)	b-ALP stable (Δb-ALP within 23%)	Decreased b-ALP (Δb-ALP > −23%)
Increased PTH (ΔPTH > + 43%)	**17 (18%)**	7 (8%)	*0 (0%)*
PTH stable (ΔPTH within 43%)	26 (28%)	**27 (29%)**	1 (1%)
Decreased PTH (ΔPTH > −43%)	*4 (4%)*	7 (8%)	**4 (4%)**
6-week (n = 93)	Increased P1NP (P1NP > + 32%)	P1NP stable (P1NP within 32%)	Decreased P1NP (P1NP > −32%)
Increased PTH (ΔPTH > + 43%)	**4 (4%)**	9 (10%)	*2 (2%)*
PTH stable (ΔPTH within 43%)	16 (17%)	**50 (53%)**	2 (2%)
Decreased PTH (ΔPTH > −43%)	*5 (5%)*	2 (2%)	**3 (3%)**
One-year (n = 93)	Increased P1NP (P1NP > + 32%)	P1NP stable (P1NP within 32%)	Decreased P1NP (P1NP > −32%)
Increased PTH (ΔPTH > + 43%)	**19 (20%)**	5 (5%)	*0 (0%)*
PTH stable (ΔPTH within 43%)	25 (28%)	**28 (30%)**	1 (1%)
Decreased PTH (ΔPTH > −43%)	*3 (3%)*	7 (7%)	**5 (5%)**
6-week (n = 93)	Increased TRAP-5b (TRAP-5b > + 24%)	TRAP-5b stable (TRAP-5b within 24%)	Decreased TRAP-5b (TRAP-5b > −24%)
Increased PTH (ΔPTH > + 43%)	**3 (3%)**	8 (9%)	*4 (4%)*
PTH stable (ΔPTH within 43%)	12 (13%)	**48 (51%)**	8 (9%)
Decreased PTH (ΔPTH > −43%)	*2 (2%)*	3 (3%)	**5 (5%)**
One-year (n = 93)	Increased TRAP-5b (TRAP-5b > + 24%)	TRAP-5b stable (TRAP-5b within 24%)	Decreased TRAP-5b (TRAP-5b > −24%)
Increased PTH (ΔPTH > + 43%)	**4 (4%)**	16 (17%)	*4 (4%)*
PTH stable (ΔPTH within 43%)	10 (11%)	**29 (32%)**	15 (16%)
Decreased PTH (ΔPTH > −43%)	*1 (1%)*	5 (5%)	**9 (10%)**
6-week (n = 93)	Increased P1NP (P1NP > + 32%)	P1NP stable (P1NP within 32%)	Decreased P1NP (P1NP > −32%)
Increased b-ALP (Δb-ALP > + 23%)	**9 (10%)**	7 (7%)	*3 (3%)*
b-ALP stable (Δb-ALP within 43%)	15 (16%)	**54 (58%)**	1 (1%)
Decreased b-ALP (Δb-ALP > −43%)	*1 (1%)*	0 (0%)	**3 (3%)**
One-year (n = 93)	Increased P1NP (P1NP > + 32%)	P1NP stable (P1NP within 32%)	Decreased P1NP (P1NP > −32%)
Increased b-ALP (Δb-ALP > + 23%)	**33 (35%)**	13 (14%)	*1 (1%)*
b-ALP stable (Δb-ALP within 43%)	14 (15%)	**25 (27%)**	2 (2%)
Decreased b-ALP (Δb-ALP > −43%)	*0 (0%)*	2 (2%)	**3 (3%)**
6-week (n = 93)	Increased TRAP-5b (TRAP-5b > + 24%)	TRAP-5b stable (TRAP-5b within 24%)	Decreased TRAP-5b (TRAP-5b > −24%)
Increased b-ALP (Δb-ALP > + 23%)	**4 (4%)**	5 (5%)	*10 (11%)*
b-ALP stable (Δb-ALP within 43%)	11 (12%)	**52 (56%)**	7 (7%)
Decreased b-ALP (Δb-ALP > −43%)	*2 (2%)*	2 (2%)	**0 (0%)**
One-year (n = 93)	Increased TRAP-5b (TRAP-5b > + 24%)	TRAP-5b stable (TRAP-5b within 24%)	Decreased TRAP-5b (TRAP-5b > −24%)
Increased b-ALP (Δb-ALP > + 23%)	**11 (12%)**	30 (32%)	*6 (6%)*
b-ALP stable (Δb-ALP within 43%)	4 (4%)	**20 (21%)**	17 (18%)
Decreased b-ALP (Δb-ALP > −43%)	*0 (0%)*	0 (0%)	**5 (5%)**
6-week (n = 93)	Increased TRAP-5b (TRAP-5b > + 24%)	TRAP-5b stable (TRAP-5b within 24%)	Decreased TRAP-5b (TRAP-5b > −24%)
Increased P1NP (ΔP1NP > + 32%)	**8 (9%)**	9 (10%)	*8 (9%)*
P1NP stable (ΔP1NP within 32%)	8 (9%)	**48 (52%)**	5 (5%)
Decreased P1NP (ΔP1NP > −43%)	*1 (1%)*	2 (2%)	**4 (4%)**
One-year (n = 93)	Increased TRAP-5b (TRAP-5b > + 24%)	TRAP-5b stable (TRAP-5b within 24%)	Decreased TRAP-5b (TRAP-5b > −24%)
Increased P1NP (ΔP1NP > + 32%)	**13 (14%)**	27 (29%)	*7 (7%)*
P1NP stable (ΔP1NP within 32%)	2 (2%)	**22 (23%)**	16 (17%)
Decreased P1NP (ΔP1NP > −43%)	*0 (0%)*	1 (1%)	**5 (5%)**

**PTH versus b-ALP (6-week)**.

Perfect concordance (bold cases): 56/93 = 60.2%.

Discordance (roman cases): 32/93 = 34.4%.

Severe discordance (italic cases): 5/93 = 5.4%.

Percentage of perfect concordance (bold cases) excluding stable patients (the central bold case, n = 53)): 3/40 = 7.5%.

**PTH versus b-ALP (one-year)**.

Perfect concordance (bold cases): 48/95 = 51.6%.

Discordance (roman cases): 41/93 = 44.1%.

Severe discordance (italic cases): 4:93 = 4.3%.

Percentage of perfect concordance (bold cases) excluding stable patients (the central bold case, n = 27)): 21/66 = 31.8%.

**PTH versus P1NP (6-week)**.

Perfect concordance (bold cases): 57/93 = 61.3%.

Discordance (roman cases): 29/93 = 31.2%.

Severe discordance (italic cases): 7/93 = 7.5%.

Percentage of perfect concordance (bold cases) excluding stable patients (the central bold case, n = 50)): 7/43 = 16.3%.

**PTH versus P1NP (one-year)**.

Perfect concordance (bold cases): 52/93 = 55.9%.

Discordance (roman cases): 38/93 = 40.9%.

Severe discordance (italic cases): 3/93 = 3.2%.

Percentage of perfect concordance (bold cases) excluding stable patients (the central bold case, n = 28)): 24/65 = 36.9%.

**PTH versus TRAP-**5b **(6-week)**.

Perfect concordance (bold cases): 56/93 = 60.2%.

Discordance (roman cases): 31/93 = 33.3%.

Severe discordance (italic cases): 6/93 = 6.5%.

Percentage of perfect concordance (bold cases) excluding stable patients (the central bold case, n = 48)): 8/45 = 17.8%.

**PTH versus TRAP-**5b **(one-year)**.

Perfect concordance (bold cases): 42/93 = 45.2%.

Discordance (roman cases): 46/93 = 49.5%.

Severe discordance (italic cases): 5/93 = 5.4%.

Percentage of perfect concordance (bold cases) excluding stable patients (the central bold case, n = 29)): 13/64 = 20.3%.

**b-ALP versus P1NP (6-week)**.

Perfect concordance (bold cases): 66/93 = 71%.

Discordance (roman cases): 23/93 = 24.7%.

Severe discordance (italic cases): 4/93 = 4.3%.

Percentage of perfect concordance (bold cases) excluding stable patients (the central bold case, n = 54)): 12/39 = 30.8%.

**b-ALP versus P1NP (one-year)**.

Perfect concordance (bold cases): 61/93 = 65.6%.

Discordance (roman cases): 31/93 = 33.3%.

Severe discordance (italic cases): 1/93 = 1.1%.

Percentage of perfect concordance (bold cases) excluding stable patients (the central bold case, n = 25)): 36/68 = 52.9%.

**b-ALP versus TRAP 5-b (6-week)**.

Perfect concordance (bold cases): 56/93 = 60.2%.

Discordance (roman cases): 25/93 = 26.9%.

Severe discordance (italic cases): 12/93 = 12.9%.

Percentage of perfect concordance (bold cases) excluding stable patients (the central bold case, n = 52)): 4/41 = 9.8%.

**b-ALP versus TRAP 5-b (one-year)**.

Perfect concordance (bold cases): 36/93 = 38.7%.

Discordance (roman cases): 51/93 = 54.8%.

Severe discordance (italic cases): 6/93 = 6.5%.

Percentage of perfect concordance (bold cases) excluding stable patients (the central bold case, n = 20)): 16/73 = 21.9%.

**P1NP versus TRAP 5b (6-week)**.

Perfect concordance (bold cases): 60/93 = 64.5%.

Discordance (roman cases): 24/93 = 25.8%.

Severe discordance (italic cases): 9/93 = 9.7%.

Percentage of perfect concordance (bold cases) excluding stable patients (the central bold case, n = 48)): 12/45 = 26.7%.

**P1NP versus TRAP 5-b (one-year)**.

Perfect concordance (bold cases): 40/93 = 43.0%.

Discordance (roman cases): 46/93 = 49.5%.

Severe discordance (italic cases): 7/93 = 7.5%.

Percentage of perfect concordance (bold cases) excluding stable patients (the central bold case, n = 22)): 18/71 = 25.3%.

### Slopes

**Figure 1:**
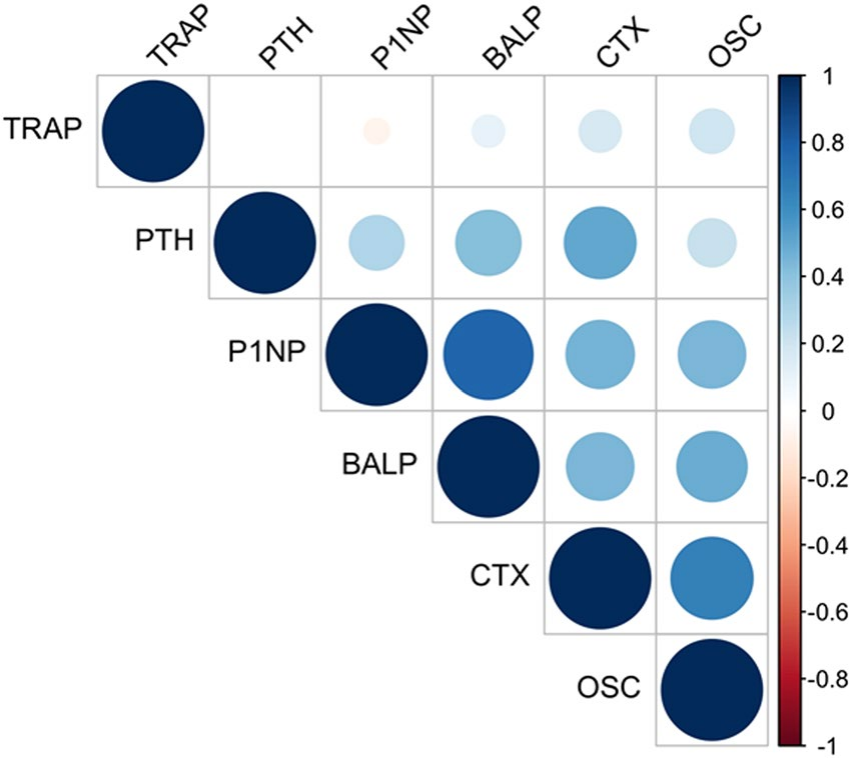
Correlation (Pearson) plot between pairs of biomarkers’ slopes calculated on 4 points. The Pearson’s coefficient is proportional to the area of each circle and the intensity of the color. The blue color represents a positive coefficient. Beta-CrossLaps, CTX, intact serum procollagen type 1 N propeptide P1NP, osteocalcin, OSC, PTH, parathormone, tartrate-resistant acid phosphatase 5B, TRAP.

Mean slopes were +0.77 ± 16.4 pg/mL/month, +1.13 ± 2.8 µg/L/month, +7.91 ± 158 ng/L/month, +5.93 ± 16.4 ng/mL/month, +4.58 ± 17.2 ng/mL/month, −0.04 ± 0.25 U/L/month/month for PTH, b-ALP, CTX, osteocalcin, P1NP and TRAP-5B, respectively. PTH slope was significantly correlated to CTX (r = 0.5, p < 0.0001), b-ALP (r = 0.41, p < 0.0001), and P1NP (r = 0.29, p = 0.004) slopes whereas no correlation was found between PTH slope and osteocalcin or TRAP-5b slopes. Apart from PTH, several pairs of biomarkers’ slopes displayed significant correlations. P1NP, b-ALP, CTX and osteocalcin slopes were significantly correlated, with a positive Pearson’s coefficient in every combination. TRAP-5B slope was only correlated to CTX. Pairs combinations are displayed in Table 5 and Fig. 1.

**Table 5:** Correlation (Pearson coefficient) matrix by pairs of biomarkers’ slopes calculated on 4 points.

	PTH	CTX	P1NP	BALP	OSC	TRAP
PTH	XXX					
CTX	*0.5* *P* < *0.0001*	XXX				
P1NP	*0.29* *P* = *0.0043*	*0.46* *P* < *0.0001*	XXX			
BALP	*0.42* *P* < *0.0001*	*0.44* *P* < *0.0001*	*0.78* *P* < *0.0001*	XXX		
OSC	0.23 P = 0.028	*0.66* *P* < *0.0001*	*0.44* *P* < *0.0001*	*0.48* *P* < *0.0001*	XXX	
TRAP	−0.01 P = 0.95	*0.17* *P* = *0.010*	−0.07 P = 0.53	0.10 P = 0.32	0.19 P = 0.06	XXX

Only the slope of TRAP is not well correlated to other biomarkers’ slopes.

## Discussion

Variations of biomarkers, more than one-point result, are recommended by the KDIGO to assess the bone turnover of hemodialysis patients^4^. Indeed, cross-sectional studies showed that the performance of PTH, bone biomarkers or even combinations of both is poor to predict the turnover determined by bone biopsy^3,12–14^. Changes in biomarkers over time are associated with mortality in dialysis patients^16,17^ and could also be better predictive of bone turnover observed by bone biopsy^3^. In the current analysis, we studied the evolution and, more specifically, the concordance between variations of biomarkers over time. If we confirmed that baseline or one-year results of PTH and bone biomarkers are highly correlated in cross-sectional analyses^3,9–11^, the correlations are much less relevant if the variations or changes of biomarkers are considered, suggesting that PTH and bone biomarkers give different information^8^. In this analysis, the variable “time” seems particularly important. Over a short period of time (6-week), no correlation can be found between PTH and bone biomarkers variations (except for CTX). The correlations between variations of PTH and bone biomarkers became however significant (except for TRAP-5b) after one year of follow-up. This point is confirmed both by considering two points comparison (baseline – 6 weeks and baseline – one year) or comparison of slopes built with four points (baseline, 6 weeks, 6 months and one year). Similarly, the correlations between bone biomarkers are stronger over one-year than 6-week period, suggesting that the variations of bone biomarkers between them and still more variations of bone biomarkers and PTH must be interpreted on a relatively long period of time.

The classification of patients as stable, increasers or decliners is based on the critical difference of biomarkers, this variable having been previously determined for PTH, b-ALP, P1NP and TRAP-5b^15^. At first glance, it could be concluded that the concordance of classifications is better over a 6-week period than over one-year of follow-up. However, it must be reminded that most patients remain stable over the 6-week period. If stable patients are excluded from the analysis, it is clear that the concordance of both decliners and increasers are higher over the one-year period.

Globally, the results support a better concordance between variations of “true” bone biomarkers (P1NP, CTX, and b-ALP,) than between PTH and bone biomarkers, which is not fully unexpected as PTH is actually an indirect bone biomarker, since it is mainly influenced by calcium concentration^8,18^. Moreover, PTH concentrations rapidly follow any modification of calcium concentration, although other bone biomarkers take more time because their concentration will depend on the bone-remodelling process, which is about six months in healthy individuals. In the same view, the half-life of bone biomarkers in serum is expressed in days, whereas the half-life of PTH is only a few minutes^8,19^. Differences between the kinetics of the “minute to minute” calcium regulation by PTH, which has a short half-life, and the time needed for bone to be altered or improved, which is sometimes over 1 year have been illustrated in peritoneal dialysis patients moving to low calcium dialysate^20^ and in hemodialyzed patients treated by cinacalcet^14,21^.

Globally, the concordances are better between bone biomarkers, with the exception of TRAP-5b. Variations of P1NP and b-ALP over time seem particularly concordant. Moreover, concentrations of these two biomarkers (at least the intact form of P1NP) are not influenced by CKD status^8,22^, contrary to CTX^3,8,23,24^. The choice of one biomarker could be dependent on the availability of the biomarker in the laboratory and/or some specific characteristics of the patients (for instance, it has been suggested that severe hepatic dysfunction could interfere with b-ALP measurement)^8,25^. Also from an analytical point of view, intact P1NP seems more robust than b-ALP^6^ and is considered as the formation marker of choice by the International Osteoporosis Foundation^26^. The lower concordance observed with TRAP-5b could be explained by the fact that this biomarker is the only bone biomarker of osteoclastic activity (or, even more, a marker of osteoclasts number), the other being markers of bone formation^8,27,28^. In this context, it must be underlined that TRAP-5b classified many patients as decliners whereas decliners are minority with P1NP and b-ALP. TRAP-5b has also be proposed as an interesting biomarker for bone mass monitoring^29^.

There are limitations to our study. First, the study is purely observational and was neither designed nor powered to determine why and for which patients’ discrepant results are observed, notably according to their therapies. Analyses were repeated after excluding 9 patients who changed their therapy (regarding cinacalcet or active vitamin D therapy) during the follow-up and the same conclusions remained (data not shown). The number of patients with changing therapy was definitively too low to be analyzed separately. A study with a similar design but focusing on patients starting cinacalcet or active vitamin D would be of interest. Second, in the absence of bone biopsy data, only a description of concordances and discrepancies in bone biomarkers evolution can be done but the potential superiority of one biomarker over another one to assess turnover cannot be proved. Third, because relative differences were used, the results could be too largely influenced by low PTH concentrations at baseline. Analyses were thus repeated excluding patients with low turnover at baseline (defined as PTH lower than two-times the upper normal value) (n = 12) and the results remained the same (see supplements), suggesting that the level of PTH at baseline did not modify the conclusions. Lastly, our follow-up remains limited (one-year) and our population contains only prevalent dialysis patients. Further analyses in incident patients and with a still longer follow-up could be of interest.

Bone biomarkers have been suggested as of interest to detect bone turnover abnormalities in dialysis patients. A dynamic view and a focus on bone biomarkers modifications is promoted^3,8^. However, as illustrated in the present study, there are frequently discrepancies between the evolution of these different markers. One can recommend that results must be analyzed on a relatively long period of time because these bone biomarkers have certainly not the same reactivity to reflect bone activity, and this is especially true comparing PTH with bone biomarker. Moreover, a better concordance is observed between “true” bone formation biomarkers than between bone biomarkers and PTH (or marker of osteoclastic activity).

## Electronic supplementary material

Dataset 1
