# Supplementary material for: Variations of parathyroid hormone and bone biomarkers are concordant only after a long term follow-up in hemodialyzed patients
Source: Sci Rep. 2017 Oct 3;7:12623. doi: 10.1038/s41598-017-12808-3 (PMC5626722; doi:10.1038/s41598-017-12808-3)
Supplement: Supplementary file 1 — Dataset 1 [file 41598_2017_12808_MOESM1_ESM.doc]

**Variations of parathyroid hormone and bone biomarkers are concordant only after a long term follow-up in hemodialyzed patients.**

**Pierre Delanaye^1^, MD, PhD, Xavier Warling^2^, MD, Martial Moonen^2^, MD, Nicole Smelten^3^, MD, François Jouret^1^, MD, PhD, Jean-Marie Krzesinski^1^, MD, PhD, Nicolas Maillard^4^, MD, PhD, Hans Pottel^5^, PhD, Etienne Cavalier^6^, EuSpLM, PhD**

**^1^ Division of Nephrology-Dialysis-Transplantation, University of Liège, CHU Sart Tilman (ULg CHU), Liège, Belgium**

**^2^ Division of Nephrology-Dialysis, Centre Hospitalier Régionale (CHR) « La Citadelle », Liège, Belgium**

**^3^ Division of Nephrology-Dialysis, Centre Hospitalier Bois de l’Abbaye et de Hesbaye (CHBAH), Seraing, Belgium**

**^4^ Division of Nephrology-Dialysis-Transplantation, Hôpital Nord, University Jean Monnet, Saint Etienne, France**

**^5^ Division of Public Health and Primary Care, KU Leuven Campus Kulak Kortrijk, Kortrijk, Belgium**

**^6^ Division of Clinical Chemistry, University of Liège (ULg CHU), Liège, Belgium**

**Supplement: Coefficient of correlation between variations (Δ) of biomarkers between baseline and one year, excluding patients with low PTH (n=12) at baseline (lower than two times the upper normal value) (n=81)**

|  | **ΔPTH** | **Δb-ALP** | **ΔCTX** | **Δosteocalcin** | **ΔP1NP** | **ΔTRAP-5b** |
| --- | --- | --- | --- | --- | --- | --- |
| **ΔPTH** | XXXXX |  |  |  |  |  |
| **Δb-ALP** | 0.30  P=0.007 | XXXXX |  |  |  |  |
| **ΔCTX** | 0.51  P<0.0001 | 0.40  P=0.0002 | XXXX |  |  |  |
| **ΔOsteocalcin** | 0.25  P=0.0234 | 0.41  P<0.0001 | 0.31  P=0.0049 | XXXXX |  |  |
| **ΔP1NP** | 0.36  P=0.0001 | 0.71  P<0.0001 | 0.47  P<0.0001 | 0.61  P<0.0001 | XXXX |  |
| **ΔTRAP-5b** | -0.08  NS | 0.06  NS | 0.22  NS | 0.22  P=0.0448 | 0.16  NS | XXXXX |

NS: not significant. Significant results in grey zones.
